# Artificial Intelligence-Assisted Heating Ventilation and Air Conditioning Control and the Unmet Demand for Sensors: Part 1. Problem Formulation and the Hypothesis

**DOI:** 10.3390/s19051131

**Published:** 2019-03-06

**Authors:** Chin-Chi Cheng, Dasheng Lee

**Affiliations:** Department of Energy and Refrigerating Air-Conditioning Engineering, National Taipei University of Technology, Taipei 10608, Taiwan; newmanch@ntut.edu.tw

**Keywords:** artificial intelligent (AI), heating ventilation and air conditioning (HVAC) system, forecasting/predicting error, priori information notice (PIN), energy management system (EMS), energy savings, normalized Harris index (NHI)

## Abstract

In this study, information pertaining to the development of artificial intelligence (AI) technology for improving the performance of heating, ventilation, and air conditioning (HVAC) systems was collected. Among the 18 AI tools developed for HVAC control during the past 20 years, only three functions, including weather forecasting, optimization, and predictive controls, have become mainstream. Based on the presented data, the energy savings of HVAC systems that have AI functionality is less than those equipped with traditional energy management system (EMS) controlling techniques. This is because the existing sensors cannot meet the required demand for AI functionality. The errors of most of the existing sensors are less than 5%. However, most of the prediction errors of AI tools are larger than 7%, except for the weather forecast. The normalized Harris index (NHI) is able to evaluate the energy saving percentages and the maximum saving rations of different kinds of HVAC controls. Based on the NHI, the estimated average energy savings percentage and the maximum saving rations of AI-assisted HVAC control are 14.4% and 44.04%, respectively. Data regarding the hypothesis of AI forecasting or prediction tools having less accuracy forms Part 1 of this series of research.

## 1. Introduction

Heating, ventilation, and air conditioning (HVAC) systems provide a suitable living environment with thermal comfort and air quality. These mechanic–electrical systems include several types, such as air conditioners, heat pumps, furnaces, boilers, chillers, and packaged systems [1]. In most of the countries, the building sector accounts for nearly 40% of the total consumed energy [2]. For every building type, HVAC and lighting systems occupy more than half of the energy consumption [3]. A large fraction of the increasing energy expenditure for the buildings was because of the extending HVAC installations for better thermal comfort and air quality [4]. Therefore, the HVAC system plays an important role in the energy efficiency of buildings. Improving the control of HVAC operations and the efficiency of the HVAC system can save significant energy, increase thermal comfort, and contribute to improved indoor environmental quality (IEQ) [5]. Artificial intelligence (AI) was founded as an academic discipline in 1956. In contrast to human intelligence, AI demonstrates machine intelligence and imitates human behaviors through mathematical coding and mechanical works. In 1997, an AI program known as Deep Blue defeated the reigning world chess champion, Garry Kasparov [6]. It was the first time that the chess-playing computer performed better than a human. That moment was a turning point in the development of AI that enabled AI to be utilized more in a wider range of applications.

In this study, how AI could improve the performance of heating, ventilation, and air conditioning (HVAC) systems was investigated. A total of 783 articles, which were related to AI research and its application on HVAC systems, was collected from three databases, including the Science Direct on Line (SDOL), IEEE Xplore (IEL Online), and MDPI. The MDPI database is a publisher of open access journals. Following the preferred reporting items for systematic reviews and meta-analyses (PRISMA) method [7] for reporting, systematic review, and meta-analysis, the collected articles were screened, and only 97 full-text articles met the requirements. All of the selected articles regard theoretical work and practical experiments about HVAC control. Detailed information of these articles, including the study cases, AI tools, or developments, and the improved performance of HVAC systems, are presented in Section 2 and summarized in Table 1. Among the 18 developed AI tools, only two methodologies have become mainstream elements of HVAC controls over the past 20 years, which are the forecasting and optimization and the predictive controls. These two main methodologies will be discussed in Section 3.

Even though the development of AI tools for HVAC systems is more than two decades old, the performance of HVAC systems controlled by AI tools has been unsatisfactory overall. Their energy savings, energy consumption, precision of heating and cooling based on load forecasting, and the predictive ability of the predictive controls, will be discussed in Section 4. Based on [8], from 1976 to 2014, the average energy savings of HVAC systems by applying the scheduling control technique reached 14.07%. The maximum energy savings of HVAC systems was 46.9% after applying smart sensors for smart air conditioners in 2014 [9]. However, from 1997 to 2018, the average energy savings of HVAC systems using AI tools reached 14.02%. The maximum energy savings when applying case-based reasoning (CBR) controlling tools for the HVAC systems in an office building was only 41% in 2014. Therefore, the energy savings of HVAC systems after applying AI tools was less than that of traditional energy management system (EMS) controlling techniques.

This study will be conducted in three parts, including (1) problem formulation and the hypothesis, (2) simulations and verification, and (3) confirmatory experiments. The first part, problem formulation and the hypothesis, will analyze the problem of HVAC systems using AI tools having less accuracy of forecasting, or and a prediction of the tools that result in poor energy savings is hypothesized. If forecast accuracies could be improved and prediction errors could be reduced, the energy savings of HVAC systems would improve. From the 35 collected articles with information regarding sensor specifications, the literature states that the existing sensors are for feedback control, not prediction, and therefore lack the capability to provide priori information notice (PIN). Hence, an innovative PIN sensor design and more precise predictive control is presented in this study as the solution to increase the energy savings of HVAC systems.

The second part of the study covers the simulation and verification of the energy-saving hypothesis and PIN sensor design through numerical simulation. Through numerical simulation, the calculated energy savings of an HVAC system using a PIN sensor will be provided. The third part consists of the confirmatory experiment where the designed PIN sensors are utilized under the various operating conditions of an HVAC system in an environmentally controlled room to measure energy consumption. The energy consumption of the HVAC system utilizing the PIN sensors and AI tools will be compared with those employing the proportional–integral–differential (PID) controllers, and the simulation results are analyzed to give evidence of the hypothesis presented in this study.

## 2. AI Developments and the Applications for HVAC Systems

In this study, keywords including AI, machine learning, heating, ventilation, and air conditioning, were utilized to conduct a paper survey from the Science Direct on Line (SDOL), IEEE Xplore (IEL Online), and MDPI databases. Initially, 737 papers were found from SDOL fitting the criteria of our paper survey, while 34 were found from IEEE Xplore, and 12 were found from MDPI. After further review, articles that were not related to HVAC control or methods to enhance performance were separated out. A total of 79 articles fit the requirements of either (1) describing the applying factory; (2) developing innovative AI tools and their use involving HVAC control; and (3) depictions describing the overall performance of an HVAC system after applying AI control tools. These articles were chosen for further exploration.

### 2.1. Study Case

The published year, HVAC system, developed AI technology, and key results of the collected 79 articles are listed in Table 1.

### 2.2. Developed AI Tools

In the second column of Table 1, there are 18 AI tools for HVAC systems. Among them, the most well-known AI tools are neuro networks (NN), including artificial neuro networks (ANN), recurrent neuro networks (RNN), spiking neuro networks (SNN), and wavelet ANN [15,16,19,22,23,24,27,29,34,35,37,39,40,44,51,52,53,59,60,64,76,79,81,82,85,87,98,99,100,102]. ANN is based on the nervous system, the human brain architecture, and the learning processes. A set of interconnected neurons can be separated into three layers, which are composed of input, output, and hidden layers. The HVAC system inputs, network weights, and the transfer functions of the network lead to the output of ANN. The ANN controller doesn’t need to identify the control model. The weight coefficient can be regulated to minimize the costs. ANN can simulate the working procedure of the human brain; therefore, it has the capability of having insight into a complex system. However, the brain-like controller has disadvantages due to having to take a lot of time for off-line training as well as requiring a large amount of data for the system to make quality predictions.

The second AI tool is used for the predictive control functions of ANN: fuzzy or model-based predictive control (MPC) [32,42,47,48,50,65,72,75,87,92,94,101,103,104]. Predictive control provides feedback of the results of the prediction to the system to allow for the adjustment of a system’s control parameters. The predictive feedback system is different from previous control systems due to the design of the feedback sensor. Collotta etc. created a non-linear autoregressive neural network auto regressive external type (NNARX-type) structure in 2014 for indoor temperature prediction [75]. In addition to enhancing the control performance, the signal of a predictive control system could be discontinuous for a non-linear system. This is different from the continuous signals that are needed for a linear system managed by a traditional PID controller, which is based on the Laplace transform and linear transfer functions. The insight ability of the ANN is similar to the human insight process, and is a smart way to improve the performance of a non-linear system commanded by predictive control.

The third type of AI tool is known as distributed AI and the multi-agent system (MAS) [20,26,31,57,58,62,66,72,73,83]. In addition to strengthening the entire performance of a system using ANN or predictive control, the subsystems, sensors, and actuators of an HVAC system are able to communicate and interact with each other and become an even more intelligent system through the use of MAS.

The fourth type of AI tool is what is known as the genetic algorithm (GA) method, which is based on biological evolution theory [14,45,54,59,61,63,74,82,93]. The GA method utilizes global non-derivative-based optimization to tune the set points of HVAC systems and meet the thermal comfort requirements without the use of a mathematical model of the system. However, the problem with the GA method is that it requires massive calculations and long run times. Therefore, the GA method might be inappropriate for the real-time operation of an HVAC system.

The fifth type of AI tools is employed for fuzzy control [21,32,33,46,51,59,102], support vector machines (SVM), and R [28,30,38,56,79,82,89]. These two AI tools have the same amount of published articles. A fuzzy logic controller (FLC) is similar to human reasoning and can be used to control a complex system by using the rules of the IF–THEN algorithm. The utilization of fuzzy logic grades and rules yields a low real-time response speed. This situation limits the application of the FLC onto HVAC systems. However, SVM and R could be used in conjunction with the FLC for data classification by finding the hard margins of various data sets to determine the proper control methodologies, modeling, or regression for decision making. This method is used mainly for analyzing huge amounts of data, modeling, and decision making, but is rarely used for HVAC system applications.

The seventh AI tools are model-based controls [10,17,18,69,91] and deep learning (DL, or reinforced learning) [36,49,88,97,98]. The model-based control models, when used with the SVM and R tools, collect and analyze data utilizing the distributed AI tool, and communicate and interact with the MAS tool. The advantage of model-based control is its predictive strategy and high capability of observation. However, the model-based control is a feedback control methodology that can only be applied to a time-independent system. It can’t solve problems within a non-linear time-variable system. A deep learning tool could determine a control strategy according to a system’s present conditions and information from previous cases through a learning process without the use of modeling. Deep learning is one of the broader machine learning methods, which is based on learning data representations, as opposed to following task-specific algorithms. The learning types are supervised, semi-supervised, or unsupervised. For an HVAC system, deep learning is a novel methodology to achieve more intelligent control.

The knowledge-based system (KBS) [11,12,13,43] is similar to the DL tool. However, the difference between them is that the DL tool is for controlling the system, and KBS is used for building various SVM and R knowledge databases. KBS could provide an optimal control strategy for various HVAC systems through the expert system. KBS and DL are mostly used for problem-solving procedures and to support human learning, decision making, and actions. Another key tool is case-based reasoning (CBR) [78]. However, there are not many published articles regarding this. CBR is able to analyze a control strategy and provide the most optimal one in conjunction with KBS or model-based control in certain cases. Nevertheless, KBS, DL, and CBR tools all need a large amount of data to learn from, and will require a lot of time to collect the control data, which will increase initial installation costs.

In addition, there are some other AI tools worth mentioning, which include: particle swarm optimization (PSO) [35,77,80] and the artificial fish swarm algorithm (AFSA) [90] for optimizing control strategies, the hidden Markov model (HMM) [70,71,89] for modeling, radial basis function (RBF) [67,68] for data collecting and analyzing, data combining technology [94,95], k-nearest neighbor (KNN) [89] for analyzing the closest data attribute, and the autoregressive exogenous (ARX) technique [65] for regression analysis with an external input and feedback control system.

### 2.3. AI Applications for HVAC Systems

The control methodologies of AI development can be observed by comparing columns one and two of Table 1, which outline the AI tools and related HVAC systems, respectively. There are four main HVAC system applications for AI tools, including (1) medium to large-scale utilities for commercial buildings [10,13,17,20,22,24,27,29,35,43,44,53,57,63,64,66,71,72,73,76,78,80,82,84,87,91,96,100,105], (2) air conditioners or chillers for residential buildings [11,15,18,19,21,36,37,38,39,42,51,52,60,61,62,65,67,68,69,70,72,75,79,83,86,88,92,94,97,98,99,101,102], (3) air conditioning systems for composite buildings [25,28,30,34,40,45,50,54,56,58,59,74,77,81,85,90,93,95,103,104], and (4) specific systems, such as a greenhouse, a regenerating power system, a power system, etc. [12,14,16,23,26].

The use of AI tools applied onto commercial and residential buildings will be discussed, due to the different occupant behavior patterns between the two building types. The occupants of commercial buildings operate within the confines of working in the numerous companies within a commercial building with a fixed office schedule, and therefore have more predictive air-conditioning demands. The HVAC systems of most commercial buildings are operated by professional energy managers under certain routines and energy-saving targets. Yet, the occupants of residential buildings, being residents, have different air-conditioning behaviors and demands. In general, the HVAC systems of most residential buildings are not operated by professional energy managers.

As mentioned in the previous section, ANN + fuzzy tools are the most widely utilized AI tools for commercial and residential buildings. The adoption ratios for these two types of buildings are 34.5% (10/29) and 24.2% (8/33), respectively. The ANN tool can imitate the operating model of the human brain to implement complex control strategies by learning and analyzing large amounts of data. This is suitable for commercial buildings due to the predictive nature of the occupants. Unfortunately, the ANN tool is not suitable for use in residential buildings. The ANN tool combined with DL, reinforced learning, or deep reinforcement learning (DFL) equips the system with the capability of feature extraction to analyze data and make control decisions, which replaces the need for a professional energy manager.

For commercial buildings, CBR and KBS tools operate alongside ANN + fuzzy tools. CBR and KBS tools can practice model base control and forecast several conditions, including weather, occupancy, and energy consumption, optimize control set points, improve the energy efficiency of an HVAC system, and ensure thermal comfort [13,22,24,27,29,35,43,44,53,64,76,78,84,100,105]. Based on the cases utilizing ANN, CBR, and KBS tools, the ability to make predictions is the most significant function of these AI tools. For residential buildings, DL, distributed AI, and MAS tools function alongside ANN + fuzzy tools. If the fundamental devices of HVAC systems are equipped with distributed AI tools for saving energy and ensuring the thermal comfort, and are able to interact with each other through an MAS tool, then predictive control and the prediction of future environmental conditions for enhancing a system’s overall performance could be achieved.

Finally, the most recent development of AI tools applied onto composite buildings is predictive control [50,103,104], which improves the control performance of an HVAC system by having the ability to make predictions. Composite building systems are a mix of residential commercial building systems.

## 3. Theoretical Analysis of AI Assisted HVAC Control

In this section, the control performance differences between typical HVAC controls and AI-assisted HVAC controls are analyzed quantitatively. The control outputs were calculated by the common analytic solutions of the AI-assisted HVAC controls in Table 1, which were then compared with those of the on–off and proportional–differential–integral (PID) controls.

### 3.1. Typical HVAC Control

**Figure 1:**
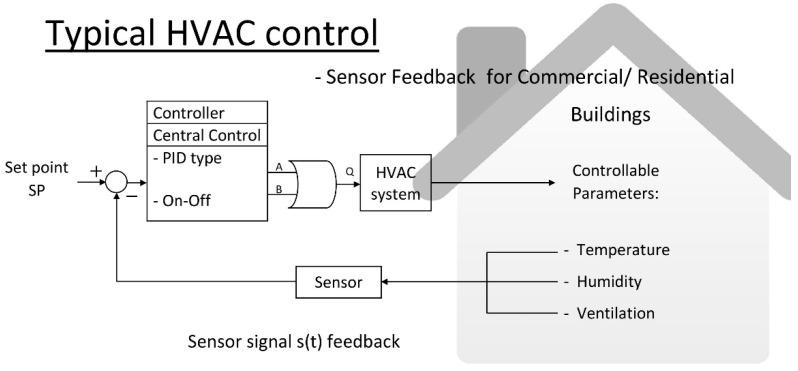
Typical HVAC controls for residential or commercial buildings.

Typical HVAC controls for residential and commercial buildings utilize on–off and PID control algorithms [106] in addition to sensor feedback controls to have the ability to control parameters such as a system’s temperature, humidity, and ventilation. The controllable structure is presented in Figure 1.

The control block diagram in Figure 1 runs PID or an on–off algorithm by comparing the set point values and sensor feedback values, and then providing the subsequent output control signals to an HVAC system.

The on–off control output values are calculated according to the following Equation:

(1) $$\sigma \left[S\left(t\right)-\mathrm{SP}\right]\{\begin{array}{ll} 1 & \mathrm{if}\;S\left(t\right)-\mathrm{SP}>Threshold\;Value\\ 0 & \mathrm{if}\;S\left(t\right)-\mathrm{SP}=0\pm \mathrm{Var}\left[S\left(t\right)\right] \end{array}$$

where σ is the step function corresponding to the difference reading of the sensor feedback, S(t), and the set point, SP, of an HVAC system. If the difference value is larger than the designed threshold value, the value of σ is one. If the difference value of S(t) and SP is within the standard variation of S(t), the value of σ is zero. The modification of on–off control is that, instead of being zero, the value of σ is located within the range of 0.5~0.7 when the difference value is within the standard variation of S(t). This is the so-called floating control to avoid the large oscillation of a control signal of the HVAC system. However, no matter how the typical on–off control or floating control is utilized, the final control signal is determined by the difference of S(t) and SP, as shown in Equation (1).

The output of PID control, as shown in Figure 1, is calculated according to the following equation:

(2) $$K_{P}\cdot \left[S\left(t\right)-\mathrm{SP}\right]+K_{I}\cdot \int \left[S\left(t\right)-\mathrm{SP}\right]\mathrm{dt}+K_{D}\cdot \frac{d\left[S\left(t\right)-\mathrm{SP}\right]}{\mathrm{dt}}$$

where $K_{P}$ is the proportional constant, $K_{I}$ is the integral constant, and $K_{D}$ is the differential constant. The differentiation between S(t)−SP is able to predict the controlling oscillation of the next stage and eliminate it within a short period. The integration of S(t)−SP is capable of providing a stable output of PID control and reaching the final state of S(t)−SP→0 after a longer period.

### 3.2. AI-Assisted HVAC Control

**Figure 2:**
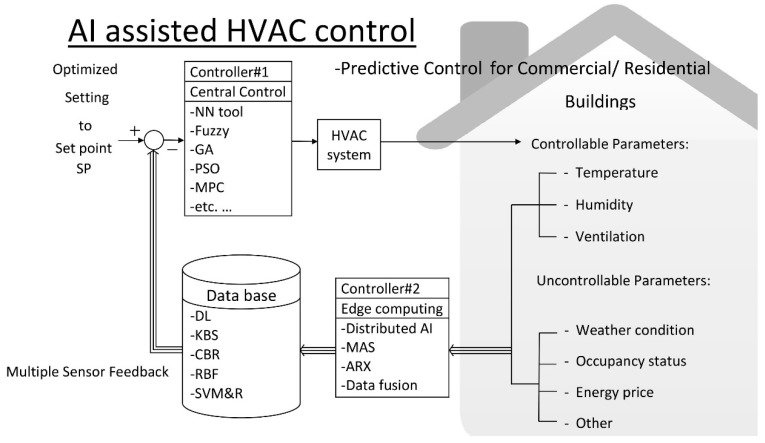
AI-assisted HVAC controls for residential and commercial buildings.

The block diagram of AI-assisted HVAC control resulting from the collected articles is shown in Figure 2.

The core of AI assisted HVAC control is the ANN tool illustrated as controller #1 in Figure 2. The output, y, of the ANN tool is produced through many processes, or neurons, and these neurons interconnect with each other by multiplying with the weights, ω, as shown in the following equation [9,15,16,22,23,24,27,29,44,47,51,52,64,75,81,85,87,99]:

(3) $$y\left(x\right)=g\left(\overset{n}{\underset{i=0}{\sum }}\omega_{i}x_{i}\right)$$

where $\omega_{0}$, $\omega_{1}$, … and $\omega_{n}$ are the weighting coefficients, and g is a non-linear activation function, which is usually a step or a sigmoid function, as illustrated by the following equation:

(4) $$g\left(x\right)=\frac{1}{1+e^{-\beta x}}\;\beta >0$$

The neuron output, y, is unidirectional both for feedback or feedforward control. The ANN tool is skilled at solving data-intensive problems within the categories of pattern classification, clustering, function approximation, prediction, optimization, content retrieval, and process control. It is similar to the human ability to make a single decision based on multiple inputs. Therefore, the main characteristic of AI-assisted HVAC control is its multiple sensor feedback, as shown in Figure 2. The multiple feedback sensor collects several sensor inputs, including controllable and uncontrollable parameters, to build a database. AI tools are not only in the central control port, as shown in controller #1 of Figure 2, but they are also applied in the sensor port, as shown in controller #2 of Figure 2, for more intelligent control.

The most utilized intelligent control functions are the optimized setting and predictive control functions, as shown in Figure 2. First, the optimized setting function utilizes the KBS [11,12,13,43,67,68,84] or CBR [34,78,105] tools from the database block to determine the set point (SP). The similarity index (SI) is employed during the calculation process, as shown in the following equation:

(5) $${\mathrm{SI}}_{i}=f\left(\left|\frac{y_{\mathrm{ic}}-y_{\mathrm{ip}}}{{\mathrm{MV}}_{i}}\right|\right)$$

where $y_{\mathrm{ic}}$ and $y_{\mathrm{ip}}$ are the neuro outputs of the variable i for the control and past case, respectively. ${\mathrm{MV}}_{i}$ is the mean difference of the variable i in the database. The function f maps the control case to the whole case difference. Based on SI, the global similarity (GS) is calculated according to the following equation:

(6) $$\;\mathrm{GS}=\frac{\sum_{i}({\mathrm{SI}}_{i}\times \omega_{i})}{\sum_{i}\omega_{i}},\;i=1,\;2,\ldots ,\;n$$

where n is the number of the controlled case and $\omega_{i}$ is the weighting coefficient.

The proportion $P_{j}$ of the prediction from the past case j is:

(7) $$P_{j}=\frac{{\mathrm{GS}}_{j}}{{\mathrm{GS}}_{T}},\;j=1,\;2,\ldots ,\;m$$

where ${\mathrm{GS}}_{T}$ is the sum of the global similarities between the selected m cases. Then, the optimized setting point (SP_opm_) can be determined by the following equation:

(8) $${\mathrm{SP}}_{\mathrm{opm}}=\;\underset{j}{\sum }\left(P_{j}\times {\mathrm{SP}}_{j}\right)/N\left(j\right)$$

where ${\mathrm{SP}}_{j}$ is the set point of past case j. The optimized set point is determined from the built database, including the previous controllable and uncontrollable parameters, and the desired SP value.

In addition to the optimized settings, other intelligent control functions are the predictive controls, which utilize the ANN + fuzzy tool as the central controller, as shown in controller #1 of Figure 2. This tool employs an IF–THEN algorithm to enhance the control performance by predicting the likelihood of future errors effectively and providing proper feedback. The SVM and R tool [28,30,38,56,79,82,89] and autoregressive with exogenous terms (ARX) tool [65] are also suitable for central and edge computing ports, respectively.

The first step of predictive control is to determine probability. After comparing the calculation methods of several articles, the suggested equation is shown in the following:

(9) Probi(t+1)=∪k∈θ[τi,k]α·[Si,k(t)]β∑k[τi,k]α·[Si,k(t)]βN(k)

where i indicates the ith sensor for detecting controllable or uncontrollable parameters. $S_{i,k}\left(t\right)$ is the ith sensor value, $\tau_{i,k}$ is the pheromone intensity, and α and β are the experience parameters. In addition to the probability value, a Guess value is also necessary for predictive control. It is calculated after the ANN runs [9,15,16,22,23,24,27,29,44,47,51,52,64,75,81,85,87,99] according to the following equation:

(10) $${\mathrm{Guess}}_{i}\left(t+1\right)=g\left(\underset{k\epsilon \theta }{\sum }\omega_{k}S_{i,k}\left(t\right)\right)$$

where $\omega_{0}$, $\omega_{1}$, … and $\omega_{n}$ are the weighting coefficients, and g is the non-linear activation function, as illustrated above. The following equation is able to predict the sensor output of the next stage.

(11) $$S\left(t+1\right)=a\cdot S\left(t\right)+b\cdot R_{1}\cdot \overset{n}{\underset{i\;=\;0}{\sum }}\mathrm{MAX}\left[{\mathrm{Prob}}_{i}\left(t+1\right)\right]+c\cdot R_{2}\cdot \overset{n}{\underset{i\;=\;0}{\sum }}{\mathrm{Guess}}_{i}\left(t+1\right)$$

where a is the momentum parameter, b is the self-influence parameter, and c is the measure insight. R_1_ and R_2_ are the random numbers within [0,1] for predictive control.

### 3.3. Control Performance Index

The Harris index (H) and normalized Harris index (NHI) [107,108] are utilized for evaluating the performances of typical and AI-assisted HVAC control outputs, as shown in the following equations:

(12) $$H=\underset{t\to \infty }{\lim}\eta_{t}=\underset{t\to \infty }{\lim}\frac{V_{1}}{\mathrm{Var}\left[y\left(t+1\right)\right]}$$

NHI = 1 − 1/H(13)

where $V_{1}=\mathrm{Var}[y|\mathrm{initial}\;\mathrm{condition}]$. The Harris index compares the variations between the initial control y(0) and y(t + 1). There are several articles discussing the effect of rising time, settling time, and overshooting [32] on the control performance of the linear system. However, the Harris index and NHI are able to assess the performance of linear, non-linear, feedforward, and feedback control systems [109], as well as thermal comfort and energy efficiency, etc.

## 4. Results and Discussions

In this study, the Harris index and NHI are employed to estimate the performance of HVAC systems in Table 1 managed by On–Off, PID, and AI-assisted control. The sensor signal outputs of the On–Off and PID controls, as shown in Equations (1) and (2), have a positive linear relationship with the Harris index. Therefore, the sensor types mentioned in the articles in Table 2 will be indicated and, then, the sensor errors will be calculated.

**Figure 3:**
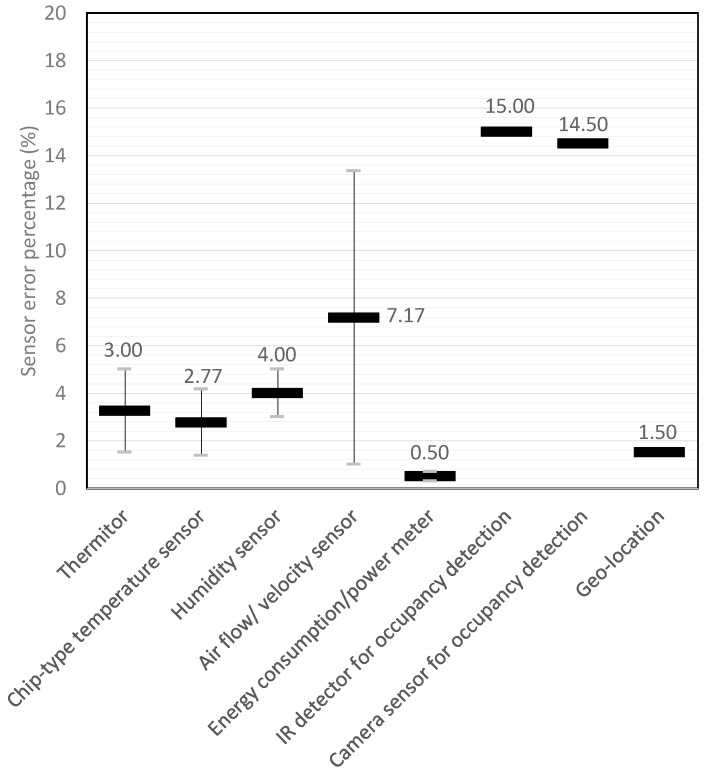
Sensor errors with respect to different type of sensors employed by AI-assisted HVAC control.

In addition, one commercialized product, Ambi Climate, with a geolocation sensor and applied sensors for the academic cases are analyzed in Table 2. The sensor types and the individual sensor errors are illustrated in Figure 3.

**Figure 4:**
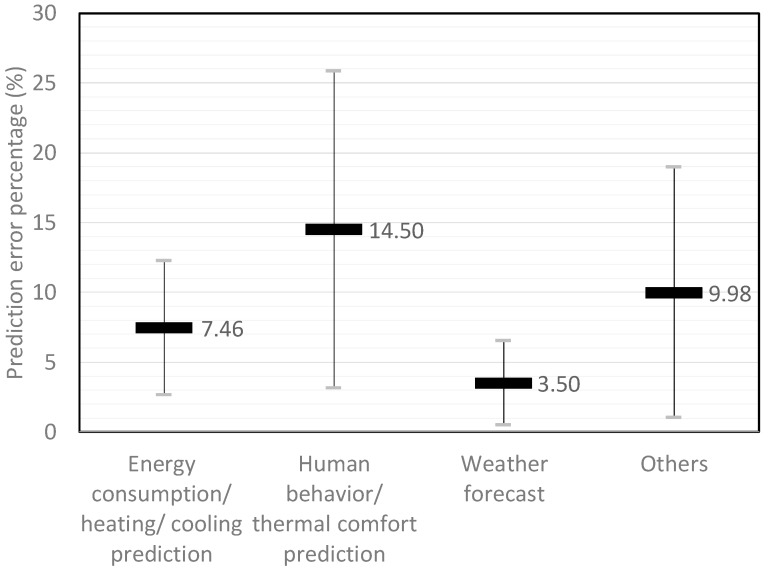
Prediction or forecast errors of AI-assisted HVAC control.

The performance indexes of On–Off and PID controls are calculated by the sensor errors, as shown in Figure 3. However, instead of sensor errors, the Harris indexes of the optimized settings and predictive controls are determined by the predictive errors, as shown in Equations (8) and (11). The collected prediction or forecast errors of AI-assisted HVAC controls in Table 1 are shown in Figure 4.

**Figure 5:**
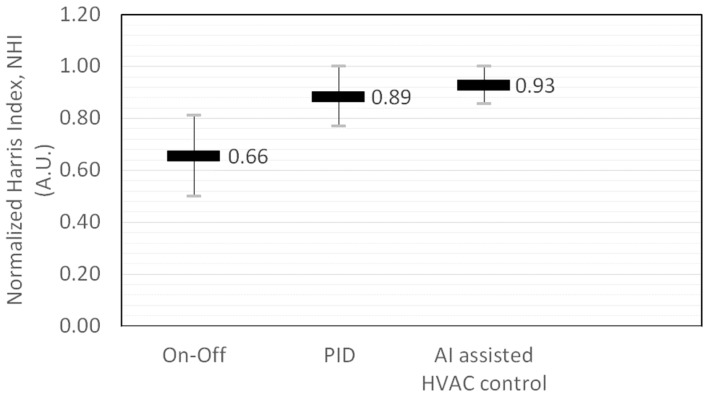
Normalized Harris index (NHI) of different kinds of HVAC controls and the expected performance improvements for energy savings.

For the On–Off control variables of $V_{1}$ and Var[y(t+1)], both are directly proportional to any sensor errors. Therefore, the calculated H is equal to one, and it becomes the comparison reference. For PID control, when the damping ratio is located in a lower damping ratio range from 0.5 to 1.5, the Var[y(t+1)] is able to reduce sensor errors by up to 30%, which will in turn enhance the H index value. Due to the reduction of the steady-state error by the integral (K_I_) control, a PID control has a better control ability than that of an On–Off control system, when the damping ratio is located within normal to lower value ranges. For higher damping ratio systems, the initial stage $V_{1}$, the final stage Var[y(t+1)], and the NHI value of the PID control will fluctuate due to variations of the proportional (K_P_) and differential (K_D_) control within a range of [0.2–0.69]. For AI-assisted HVAC control, the Var[y(t+1)] is estimated from the sensor output S(t+1), and the assumption is that the NHI is equal to one, as illustrated in Equations (12) and (13). However, the prediction or forecast errors of the AI controls fluctuate at certain ranges and cause variations of the NHI. This occurs particularly when the AI control utilizes human behavior algorithms or thermal comfort prediction algorithms, and the NHI is even lower than that of the PID control. The NHIs of On–Off, PID, and AI-assisted HVAC controls are shown in Figure 5.

**Figure 6:**
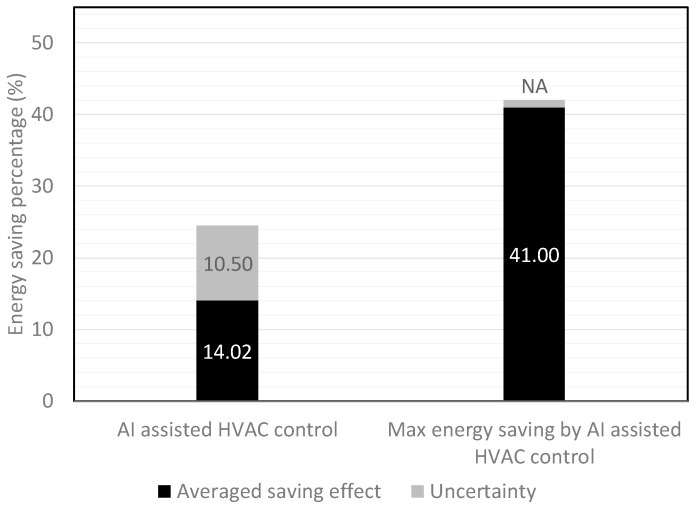
The average energy savings of the 24 cases and the maximum energy savings achieved by AI-assisted HVAC control.

The NHI is utilized to evaluate the performance of the control tools, and especially focuses on the energy-saving percentages, because of its capability to estimate the performance of linear and non-linear control systems. In Table 1, there are only 24 cases [11,12,14,19,21,44,50,57,58,62,63,72,73,83,84,92,93,94,97,98,101,103,105] that have references to the energy-saving percentages of AI-assisted HVAC controls. The average energy saving percentages of these 24 cases are shown in Figure 6, and a maximum energy savings of 41% is achieved by decision making through the MAS and CBR tools.

In Figure 6, the average energy savings percentage when using AI-assisted HVAC control is 14.02%. Of the 24 cases, 83% were comprised of On–Off control, and 17% were comprised of PID control. Based on the NHI, the estimated average energy savings percentage, variations in energy savings, and the maximum energy savings of AI-assisted HVAC control are 14.4%, 22.32%, and 44.04%, respectively. Comparing these results with the experimental data of 14.02%, 24.52%, and 41.0% in Figure 6, the errors are 3%, 9%, and 7%, respectively.

## 5. Conclusions

The presented NHI in this research can be used to evaluate the performance of AI-assisted HVAC control effectively, especially for non-linear control systems assisted by the optimized setting with CBR or KBS tools, or predictive control with the distributed AI and fuzzy algorithm. In order to calculate the NHI, the following hypotheses are made:

(1) If the prediction/forecast accuracy could reach 3.5%, which approaches the thresholds of weather forecast accuracy and the accuracies of several types of sensors, including the thermistor, chip type temperature sensor, and humidity sensor, the performance of AI-assisted HVAC control will be enhanced. When compared with the On–Off and PID control strategies, the performance of the AI-assisted HVAC control had an increase of 57.0% and 44.64%, respectively. The increased energy saving percentages are above the average, and even above the maximum energy savings that were found in any of the published articles from 1997 to 2018.
(2) In this study, the lower accuracy of the prediction tools and the resulting poor energy savings of HVAC systems are hypothesized. This hypothesis is from the collected articles, and forms the qualitative research in this paper. In the future, based on the hypothesis, the performance improvement of AI-assisted HVAC control will depend on the prediction accuracy of the sensors, which will be evidenced through the numerical simulation in Part 2 and the confirming experiments in Part 3.
(3) The existing sensors are designed for accurate sensing, but not for accurate prediction, and this causes an unmet demand of the sensors. Improved sensors for AI-assisted HVAC controls should be able to provide the ability of more accurate prediction. Based on Bayes’ theorem, accurate prediction depends on the conditional probability. The priori probability can be utilized to determine the posterior possibility, and the consistent prediction can be achieved by aggregation. The priori information notice (PIN) design for sensors are provided in this study to decrease the prediction errors to as low as 3.5% or less. The details of the PIN sensor will be discussed in Part 2 of the serial research.

## Figures and Tables

**Table 1:** Artificial intelligence (AI) developments for heating, ventilation, and air conditioning (HVAC) systems and the obtained key results.

Year	HVAC System	AI Development	Key Results	Ref.
**1997**	Case #1. A medium-sized utility from the Midwestern United States (US); Case #2. A large utility from the Midwestern US	Operation decision environment (ODE) architecture	Model-based control and fault diagnosis	[10]
**1997**	HVAC system for occupant comfort and efficient running costs	Knowledge-based system (KBS) for predictive control	Based on pre-programmed load priorities, 20% electricity savings was achieved	[11]
**1998**	MACQU software applied to a greenhouse	Native fuzzy KBS at the supervisory level	Control loop optimization and 12% energy savings	[12]
**1998**	Expert system in commercial buildings	KBS for energy conservation programs	Cost savings up to 60%	[13]
**2000**	HVAC system with variable air volume (VAV) coils and constant air volume (CAV) coils	Genetic algorithm (GA), cost estimation and model-based predictor	Simulation results show that the overall energy savings were 0.1%, 0.2%, 1.8% and 1.9% less than the original status	[14]
**2001**	Prediction of heating and cooling loads at residential buildings	Static neuro network (SNN) development for prediction	Load curve fitting with an R-square value up to 0.9887 Prediction error ranges from 2.5% to 8.7%	[15]
**2001**	Use of artificial neuro networks (ANNs) in solar radiation and wind speed prediction, photovoltaic systems, building services, and load forecasting and prediction	ANNs for modeling a solar steam generator, modeling of solar domestic water heating systems, and forecasting the building thermal loads	R-square value of load fitting ranges from 0.9733 to 0.9940. The prediction errors are within 1.9–5.5%.	[16]
**2001**	Optimal heating control of a passive solar commercial building	Smart heating controller with the cost function can combine comfort level and energy consumption	Energy savings of maintaining or improving a thermal comfort are about 9%	[17]
**2002**	House_n demonstration at Massachusetts Institute of Technology	Saving energy, maintaining air quality and thermal comfort using data analysis	Energy savings are about 14%	[18]
**2002**	SNN for analyzing energy consumption in residential buildings	Model-based control for energy savings	Energy savings range from 5% to 15%	[19]
**2003**	Building automation and energy management using AI	Distributed AI development for demand-side management (DSM) and scheduling energy consumption according to energy tariff	DSM-abled devices can save up to 40% on energy costs based on 24-h analysis	[20]
**2003**	Fuzzy controller for the management of an indoor environment	Five fuzzy controllers include fuzzy P, fuzzy proportional–integral–differential (PID), fuzzy PI, fuzzy PD and adaptive fuzzy PD	While maintaining predicted mean vote (PMV) within 0–0.1 and indoor CO_2_ ppm increased less than 20 ppm, fuzzy P controller had the best performance, heating and cooling energy can be reduced up to 20.1%.	[21]
**2003**	ANNs in the optimal operation of HVAC equipment	ANN was developed for predicting the optimal start times of a heating system in a building	In 27 instances, a clear linear relationship between prediction and real data was shown by the R-square values ranging from 0.968 to 0.996.	[22]
**2004**	ANNs for load forecasting of Taiwan power system	An integrated, evolving fuzzy neuro network and simulated annealing (AIFNN) developed for load forecasting	Compared with traditional ANNs, AIFNN can reduce prediction errors up to 3%	[23]
**2005**	On-line building energy consumption prediction through adaptive ANN	Adaptive ANN model fits the unexpected pattern changes of the incoming data of chillers at a Laval building operated from 7:30 to 23:00, Monday to Friday	The prediction accuracy is measured by the coefficient of variation (CV) and the root mean square error (RMSE). For the Laval building case, the CV is 0.20 and the RMSE = 27.0 kW. With respect to the total power consumption ~180 kw, the prediction error is 15%.	[24]
**2005**	Energy forecast of intelligent buildings located at US and United Kingdom (UK)	Increased return on investment (ROI) by using fuzzy multi-criteria decision-making method (DMM)	3% cost savings can be achieved with AI-assisted decision making.	[25]
**2005**	Adaptive control of home environment (ACHE) at Colorado	Distributed AI development and integrated with sensors	Sensors of electrical consumption with ANN adapt to the habits of inhabitants	[26]
**2005**	Predicting hourly energy consumption in buildings	ANN development for predicting short-term energy consumption and feedback control	Feedback ANN for highly efficient energy supply	[27]
**2005**	Prediction of building energy consumption in tropical regions	Support vector machine (SVM) development for accurate prediction based on weather forecast data	Summertime energy consumption can be accurately predicted within an error rate of less than 4.5%	[28]
**2005**	Prediction of daily heating loads of UK buildings	SNN development for daily heating load predictions based on one year of sensor data	Prediction error rate of less than 3.0%	[29]
**2006**	Electric load forecasting through the use of data from the East-Slovakia Power Distribution Company	SVM model development for the forecasting of a test set in January 1999	Mean average percent error (MAPE) rate of 1.93%	[30]
**2006**	Centralized HVAC system	Multi-agent structure development for thermal comfort control	Control accuracy of around 89% to 92.5%. That indicates a 7.5–11% prediction error rate related to occupants’ thermal comfort levels.	[31]
**2006**	Predictive control system development for a building heating system	Fuzzy + proportional–integral–differential (PID) controller development for improving control performance	For a heater control, temperature increase times can be reduced from 12.7 sec to 4.3 sec; the settling time can be reduced from 16.3 sec to 6.9 sec; overshooting can be reduced to 0%.	[32]
**2006**	Indoor thermal comfort controller development	Fuzzy logic controller development	The measuring period was from 15 September 2004 until 17 September 2004 at a 2-sec sample rate. The indoor air quality was kept between 600–800 ppm. The predicted mean vote (PMV) fluctuates around one	[33]
**2006**	Cooling prediction of an existing HVAC system in China	Combination of rough set (RS) theory and ANN for cooling load prediction	The HVAC system has 11 air-handling units (AHU) and operates 24 h a day. The prediction error rate of cooling energy during a 24-h period in summer time ranged from 3.45% to 9.27%	[34]
**2007**	Hourly load demand forecast	Combining evolutionary program (EP) and particle swarm optimization (PSO), combined with an artificial neural network (CANN) was developed for short-term hourly load forecasting	Hourly loads of a 6000-kW utility were predicted during the first week of December 2005. Using the best trained CANN tool, MAPE can reach 2.24% to 3.25%.	[35]
**2007**	Achieving thermal comfort in two simulated buildings	Development of a linear reinforcement learning controller instead of using a traditional on/off controller	Controller development for saving energy while maintaining thermal comfort; over a period of four years, the annual energy consumption increased marginally from 4.77 MWh to 4.85 MWh. However, the dissatisfaction index, predicted percentage of dissatisfied (PPD), was decreased from 13.4% to 12.1%.	[36]
**2008**	Forecasting building energy consumption based on simulation models and ANN	Comparison between detailed model simulations and ANN for forecasting building energy consumption	Difference between the detailed model and ANN is less than 2.1%	[37]
**2008**	Predicting monthly heating loads of residential buildings	Regression model development for prediction	MAPE ranges from 2.3% to 5.5%	[38]
**2008**	Heat load prediction of a district’s heating and cooling system	Recurrent neural network (RNN) development for heating load prediction	During a four-month period in winter, daily prediction errors rates ranged from 5.3% to 15.5%	[39]
**2009**	Year-round temperature prediction of the southeastern United States	Ward-type ANNs development for the prediction of air temperature during the entire year based on near real-time data	Using detailed weather data collected by the Georgia Automated Environmental Monitoring Network, ANNs were trained to provide prediction throughout the year. The prediction mean absolute error rate (MAE) ranged from 0.516 °C to 1.873 °C	[40]
**2009**	Measuring the prediction performance of a wet cooling tower	ANN development for the prediction of cooling tower approaching temperatures	The prediction means square error rate (MSE) of around 0.064 °C	[41]
**2009**	Control performance improvement of a typical AHU variable air volume (VAV) air-conditioning system	Model-based predictive control (MPC) development based on a first-order plus time-delay model	For an air-conditioned area of about 1200 m^2^ in Hong Kong, cooling air can track the set point with an error rate of around 0.13 °C	[42]
**2010**	KBS applications in smart homes	Autonomous caretaker to create an environmentally-friendly and comfortable ambience	Smart home ontology has the potential to save on labor costs	[43]
**2010**	A chiller system in an intelligent building	Optimization by RNN	7.4% energy savings	[44]
**2010**	Intelligent multi-player grid management for reducing energy cost	Evolutionary computation development for cost saving	1 kwh of energy cost can be reduced from 0.773 € to 0.313 €. Cost saving is around 62.4%	[45]
**2010**	Fuzzy logic controller for greenhouse applications	Fuzzy controller design for universal purpose	The controller can be used in any cultivation with different environmental variables’ set points.	[46]
**2010**	Prediction of heating energy consumption in a model house at Denizli, Turkey	Model-based prediction	Prediction errors range from 2.3% to 5.5%	[47]
**2010**	Prediction of annual heating and cooling loads for 80 residential buildings	Model-based prediction	Prediction errors range from 7.5% to 22.4%	[48]
**2011**	Adaptive learning system at intelligent buildings	Smart scheduling control based on deep learning	1.33 °C shift close to occupants’ custom settings	[49]
**2011**	Hybrid controller for energy management at a simulated one-floor building of 128 m^2^, with a bay window at the University of Perpignan Via Domitia, south of France	Fuzzy-PID schema development for model predictive control (MPC)	While maintaining thermal comfort, 1 °C exceeding the set point can be controlled to save 6% energy, but occupants will feel warm. PMV can be ensured by an 0.2 °C temperature increment. The energy saving is less than 0.3%	[50]
**2011**	Predicting air outlet temperature of an indirect evaporative cooling system	Soft computing tools include the fuzzy interference system (FIS), ANN, and adaptive neuro fuzzy inference (ANFIS)	ANN trained by the Levenbergy–Marquardt algorithm provides the best prediction performance. R^2^ value can be as high as 0.9999. Predicted temperature deviation is less than 1 °C, and the error ranges from 1.1% to 3.2%	[51]
**2011**	AI-based thermal control method for a typical US single family house	ANFIS development and the control performance comparison with ANN	ANFIS control can save 0.3% more energy than the ANN in the winter. In the summertime, ANFIS can save 0.7% more energy	[52]
**2011**	Predicting temperature and power consumption of a district boiler	Wavelet-based ANN development for accurate prediction	Prediction errors range from 4.17% to 9.01%	[53]
**2011**	Controller development for a heating and cooling system	GA-based fuzzy PID controller development	Lowering equipment initial and operating cost up to 20%	[54]
**2011**	Mining building performance data for energy-efficient operation	Energy-efficient mining model development for predicting environmental variables	The model is used to predict the environmental variables of a 4500 m^2^ south-facing low-energy building consisting of 70 rooms. The confidence of room temperature prediction is 84.63%; that of radiant temperature prediction is 90.34%; the CO_2_ concentration prediction confidence is 64.68%; and that of relative humidity is 86.76%	[55]
**2011**	Regression model development for predicting heating and cooling loads of buildings in different climates	Principal components analysis (PCA) development for predicting outdoor temperature	Prediction errors range from 5.5% to 7.9%	[56]
**2012**	Intelligent energy management system (EMS) for smart offices	Distributed AI development for optimized scheduling control of office equipment	12% energy saving	[57]
**2012**	Cloud-based EMS and future energy environment	Distributed AI and machine to machine (M2M) communication development	22.5% energy saving	[58]
**2012**	Zone temperature prediction in buildings	Predicting indoor temperature by traditional thermal dynamic model, ANN, GA, and fuzzy logic approaches	MAE of prediction by traditional model is 0.422 °C; ANN is 0.42 °C; GA is 0.753 °C, and fuzzy logic is 0.741 °C	[59]
**2012**	Forecasting household electricity consumption	RNN development for the short-term (one hour ahead) forecasting of the household electric consumption	The house is located in a suburban area in the neighbors of the town of Palermo, Italy. The prediction errors range from 1.5% to 4.6%	[60]
**2012**	Model-based control of a HVAC system in a single zone of a building	Multi-objective GA development for predicting air temperature and relative humidity	MAE of temperature prediction is 0.1–0.6 °C. Relative humidity is 0.5–3.0%	[61]
**2012**	Coordinating occupants’ behaviors for building energy and comfort management	Distributed AI development to achieve multi-agent comfort management	Reducing 12% energy consumption while keeping thermal comfort with the variation less than 0.5%	[62]
**2012**	Optimization of chiller operation at the office building of the company Imel in New Belgrade	GA development for the optimization of chiller operation	2% energy saving during warmest summer days, and up to 13% during the transition period at lower average external temperatures	[63]
**2012**	Energy efficiency enhancement of a decoupled HVAC system	Wavelet-based ANN development for optimization of scheduling control	In mid-season operation, daily operation cost can be saved from 5.88% to 11.16%	[64]
**2012**	Hourly thermal load prediction	Autoregressive with exogenous terms (ARX) model development for thermal load prediction	MAPE ranges from 9.5% to 17.5%	[65]
**2013**	Multi-agent system (MAS) application in a commercial building owned by Xerox Palo Alto Research Center (PARC) in the US	MAS development for constructing a building comfort and energy management system (BECMS)	Constructing a hierarchical function decomposition to provide user solution	[66]
**2013**	Three typical residential buildings with 3.3-kWp photovoltaic (PV) plant located at Ripatransone (AP), Italy	Radial basic function (RBF) network development for monitoring home loads, detecting and forecasting PV energy production and home consumptions, informs and influences users on their energy choices	MAPE of home load prediction for next three hours is 9.70%, eight hours is 12.20%, and 18 h is 16.30%. MAPE of PV energy production for the next three hours is 7.70%, eight hours is 9.30%, and 18 h is 11.80%.	[67] [68]
**2013**	Smart homes in a smart grid	Supervisory control and data acquisition + house intelligent management system = SHIM for charge and discharge of the electric or plug-in hybrid vehicles, and the participation in demand response (DR) programs	Considering the energy consumption data of a Portuguese house over 30 days in June 2012, the energy cost can be saved up to 12.1%	[69]
**2013**	Designing customized energy service based on disaggregation of heating usage	Estimating heat usage by hidden Markov model (HMM)	Heating usage can be predicted, and the errors range from 4.64% to 8.74%	[70]
**2013**	Using sensors commonly installed in office buildings to recognize energy-related activities	Layered HMM development for recognizing occupants’ behaviors	People counting can have the accuracy of 87% in the single-person room and 78% in the multi-person room. The away and present activity can be identified with the accuracy of 97.7% in the single-person room, but only 61% accuracy can be achieved in the multi-person room. The prediction of other activities has accuracy ranges from 98.7% to 61%	[71]
**2013**	MAS for BECMS based on occupants’ behaviors	User-oriented control based on behavior prediction	Indoor thermal comfort is considered to be highly satisfactory to occupants while maintaining a PMV of around 0.6065	[72]
**2013**	Predictive control of vapor compression cycle system	MPC development for multi-variable control	Energy saving by MPC can reach 25.31%. With the prediction by AI, energy cost can be reduced up to 28.52%. Comparing the traditional prediction by linear regression, energy-saving performance is improved by 65.53% and cost-saving can be increased up to 63%	[72]
**2013**	A survey of energy-intelligent buildings based on user activity	MAS for gathering real-time occupancy information, predicting occupancy patterns and decision making	Energy saving of HVAC equipment can reach 12%	[73]
**2013**	Optimal energy management by load shift	GA development for load shift control	35% load shift is possible under a reasonable storage capacity	[74]
**2014**	Dynamic fuzzy controller development to meet thermal comfort	ANN performs indoor temperature forecasts to deed a fuzzy logic controller	Thermal comfort is very subjective, and may vary even in the same object	[75]
**2014**	Electricity demand prediction of the center of investigation on energy solar (CIESOL) bioclimatic building	Short-term predictive neural network model development	With a short-term prediction horizon equal to 60 min, the mean error is 11.48%	[76]
**2014**	An autonomous hybrid power system	PSO development for predicting weather conditions	Techno-socio-economic criterion for the optimum mix of renewable energy resources	[77]
**2014**	Energy consumption prediction of a commercial building that has a total floor area of 34,568 m^2^ and is located in Montreal, Quebec	Case-based reasoning (CBR) model development for predicting following three-hour weather conditions and indoor thermal loads	During occupancy, 07:00–18:00, the coefficient of variation of the root mean square error (CV-RMSE) is below 13.2%, the normalized mean bias error (NMBE) is below 5.8%, and the root mean square error (RMSE) is below 14 kW	[78]
**2014**	Simulated 12 building types have the same volume, ~771.75 m^3^	SVR + ANN development for predicting heating and cooling loads with eight input parameters	Prediction error is less than 4%. Compared with the traditional model prediction, the SVR + ANN model can improve the prediction error by 39.0%	[79]
**2014**	Intelligent energy management at 45 bus stations at Alexandria	PSO development for occupancy prediction and the control of renewable energy sources	During four-hour operation, power imported from the grid can be limited by only 42%	[80]
**2014**	93 households in Portugal	ANN development for energy consumption and load forecasting	MAPE is 4.2%	[81]
**2014**	AI development for estimating building energy consumption	GA, ANN, and SVM development for building estimation models	Peak difference in hourly prediction of different models can be as high as 90%. Monthly prediction is 40% and annual variation is 7%	[82]
**2014**	Energy management optimization of a building that has wooden external walls of 9 cm and a wooden external roof of 9 cm.	Distributed AI development	Distributed AI in the end control devices can save up to 39% energy through the generation of optimal set points	[83]
**2015**	Real-world application for energy savings in a smart building at a Greek university	Rule-based approach development for optimized scheduling control	Daily energy saving can reach up to 4%	[84]
**2015**	100 load curves in a smart grid	ANN development for DSM	Prediction error is less than 5.5%	[85]
**2015**	Five AI algorithms conducted in a one-story test building with a double skin; the building is 4.2 m wide, 4.5 m deep, and 3.05 m high.	AI theory-based optimal control algorithm development for improving the indoor temperature conditions and heating energy efficiency	Compared with the transitional algorithm, this novel algorithm can increase thermal comfort by around 2.27%	[86]
**2015**	Solar combi-system combined with a gas boiler or a heat pump	ANN model development for predicting thermal load	Based on a learning sequence lasting only 12 days, the annual prediction errors are less than 10%	[87]
**2015**	Home energy management system in 25 households in Austria	Short-term smart learning electrical load algorithm development to increase flexibility to fit more the generation from renewable energies and micro co-generation devices	Prediction error is less than 8.2%	[88]
**2015**	Three houses with wireless sensors for detecting use occupancy and activity patterns	Non-linear multiclass SVM, HMM, and k-nearest neighbor (kNN) model development to deal with the complex nature of data collected from various sensors	AI algorithm development can increase 25% performance for predicting occupants’ behaviors	[89]
**2015**	Modeling for smart energy scheduling in micro-grids	Operation policy and artificial fish swarm algorithm (AFSA) for suggesting operation policy (scheduling control) of a micro-grid with V2G (Vehicle to Grid)	5.81% energy cost saving	[90]
**2016**	Hybrid renewable Energy systems	AI development for tariff control	10% reduction of unit energy price	[91]
**2016**	Model-based predictive control for building energy management	Model-based predictive controller development	Set point optimization by occupants’ activities can save 34.1% energy	[92]
**2016**	Multi-objective control and management for smart energy buildings	Hybrid multi-objective GA development	31.6% energy savings can be achieved for a smart building. Compared with traditional optimization methods, thermal comfort can be improved by 71.8%	[93]
**2016**	Hot water demand prediction model development for residential energy management systems	Bottom–up approach development	Total energy savings of 18.25%. Among them, 1.46% of that is attributed to the use of AI tools, compared with linear-up prediction.	[94]
**2016**	Hybrid forecasting model based on data preprocessing, optimization, and AI algorithms	AI-assisted data fusion	MAPE ranges from 4.57% to 5.69%	[95]
**2017**	Estimation of the energy savings potential in national building stocks	AI for analyzing user behaviors	User-behavior trends were taken into account and up to a 10% improvement of prediction accuracy resulted	[96]
**2017**	Deep reinforcement learning for building HVAC control	Deep reinforcement learning (DFL)-based algorithm	11% energy savings	[97]
**2017**	Office heating ventilation and air conditioning systems	Reinforcement learning (RL) and long/short-term memory RNN	2.5% energy savings while improving thermal comfort by an average of 15%	[98]
**2018**	Manager’s decision-making system for household energy savings	ANN-based decision making system (DMS) development	Electricity bills could be reduced by around 10%	[99]
**2018**	Energy consumption forecasting for building energy management systems	Elman neuro network	Mean square error rate (MSE) ranges from 0.004413 to 0.005085	[100]
**2018**	Home air conditioner energy management and optimization strategy with demand response	MPC for demand response and air conditioning control	9.2% energy savings when compared to conventional On/Off control and 1.8% energy savings compared with PID control	[101]
**2018**	Non-linear control techniques for HVAC systems	Fuzzy control	Smoothly reaches to set point values. The steady state error rates range from 0.2% to 3.3%	[102]
**2018**	Enhancing building and HVAC system energy efficiency	MPC	Most cases have an energy-savings rations range from 10% to 15%	[103]
**2018**	Building air conditioning systems in micro-grids	Distributed economic model predictive control (DEMPC)	Predictions of energy prices are within 3%	[104]
**2018**	HVAC systems at an office building	MAS and CBR for energy management and decision making	41% energy savings	[105]

Table 1 lists all the articles related to the application of AI technologies on the HVAC systems from 1997 to 2018 according to the PRISMA method. The results of the qualitative analysis of Table 1 are described in the following sections.

**Table 2:** Different sensors employed by AI-assisted HVAC control.

**Year**	**Academic Case**	**AI Application Scenario**	**Sensor Deployment**	**Ref.**
**1997**	Heating, ventilation, and air conditioning (HVAC) system for improving occupant comfort and saving running costs	Optimized setting	CO_2_ sensor Fire sensor Occupancy sensor Temperature sensor	[11]
**2000**	HVAC system with variable air volume (VAV) coils and constant air volume (CAV) coils	Predictive control	CO_2_ sensor Flow rate sensor Pressure sensor Humidity sensor Temperature sensor Volatile organic compounds (VOCs) concentration sensor	[14]
**2001**	Optimal heating control in a passive solar commercial building	Optimized setting	Thermal comfort sensor module includes the ambient temperature sensors and solar radiation sensors Water temperature sensor Energy consumption meter	[17]
**2002**	House_n demonstration at Massachusetts Institute of Technology	Optimized setting	A fixed, wide-color camera, a microphone, and a temperature sensor	[18]
**2003**	Fuzzy controller development for energy conservation and users’ indoor comfort requirements	Fuzzy control for improving control performance	Hybrid sensor module consists of temperature humidity, air velocity, CO_2_, mean radiant temperature gauge, etc. Outdoor temperature and humidity sensors Indoor illuminance sensor Indoor temperature sensor Power meter	[21]
**2003**	Artificial neuro network (ANN) development for optimal operation of heating system in building	Predictive control	Simulation based on temperature sensor data, thermal resistances, and indoor heat gains	[22]
**2005**	Predicting chiller energy consumption at a Laval building operated from 7:30 to 23:00, Monday to Friday	Model-based predictive control	Outdoor dry-bulb temperature sensor Wet-bulb temperature sensor Horizontal solar flux sensor Status detector of chiller Water temperature sensor Flow meter Electric power meter	[24]
**2005**	Internet-based HVAC system allows authorized users to keep in close contact with a building automation system	Optimized setting	Web-enabled controller with pressure, temperature, and flow sensors	[25]
**2006**	Centralized HVAC system with multi-agent structure	Distributed AI	Simulation based on thermal comfort related sensors	[31]
**2006**	Predictive control system development for a building heating system	Predictive control	Temperature sensor	[32]
**2006**	Indoor thermal comfort controller development	Fuzzy indoor thermal comfort controller development by simulation software	Simulation based on inputs from light sensor, outdoor temperature sensor, relative humidity sensor, air flow/hotwire anemometer, and CO_2_ sensor	[33]
**2006**	Cooling load prediction of an existing HVAC system in China	Load prediction	Multiple sensor data input includes temperature, relative humidity, and pressure	[34]
**2007**	Linear reinforcement learning controller	Machine learning and the adaptive occupant satisfaction simulator	Three different configurations include: Indoor temperature; outdoor temperature; relative humidity; CO_2_ Indoor temperature; outdoor temperature; time; CO_2_ Indoor temperature; outdoor temperature; CO_2_	[36]
**2008**	Heating load prediction of a district heating and cooling system	Load prediction	Temperature sensor Weather meter	[39]
**2009**	Controller development for a typical variable air volume (VAV) air conditioning system	Model-based predictive control	Pressure sensor Temperature sensor Humidity sensor Flow station CO_2_ sensor	[42]
**2010**	Chiller development for an intelligent building	Predictive control and optimized setting	Temperature sensor Power meter	[44]
**2011**	Controller development for air conditioning system of one-floor building	Fuzzy PID	Temperature sensor Relative humidity sensor Solar radiation sensor Power meter	[50]
**2011**	Thermal control of a typical US single family house	Fuzzy logic and adaptive neuro fuzzy inference system (ANFIS)	Temperature sensor	[52]
**2011**	Controller development for a heating and cooling energy system	Predictive control	Temperature sensor	[54]
**2012**	Zone temperature prediction and control in buildings	Predictive control and optimized setting	Chilled water valve opening level Chilled water flow rate sensor Chilled temperature sensor Outdoor temperature sensor Indoor temperature sensor	[59]
**2012**	Model-based predictive control of HVAC systems for ensuring thermal comfort and energy consumption minimization	Predictive control and optimized setting	Wireless sensor network with activity detector, temperature sensor, humidity sensor, mean radiant temperature sensor, doors/windows state detector Weather station includes solar radiation, temperature, and relative humidity	[61]
**2012**	Coordinating occupant behavior for saving energy consumption of an HVAC system and improving thermal comfort	Distributed AI	Real-world feedback data Building/occupant data Occupant suggestions	[62]
**2012**	Optimization of chiller operation at the office building of the Imel company in New Belgrade	Optimized setting	The outlet temperature from the chiller (evaporator outlet temperature sensor) The return temperature sensor The external temperature sensor	[63]
**2012**	Energy-efficiency enhancement of decoupled HVAC system	Wavelet-based artificial neuro network (WNN)—Infinite impulse response (IIR)—PID-based control	Temperature sensor Humidity sensor Air flow meter Water flow meter	[64]
**2013**	Building energy and comfort management system development	Distributed AI	Sensors provide Environmental data Occupancy data Energy data	[72]
**2013**	Energy intelligent building based on user activity	Distributed AI and predictive control	Wireless sensor networks include PIR sensors and magnetic reed switch door sensor	[73]
**2013**	Predictive control of a cooling plant	Model-based predictive control	Temperature sensor	[71]
**2014**	Dynamic fuzzy controller	Predictive control	ANN forecasted parameters	[75]
**2014**	Energy management optimization of a building	Distributed AI	Indoor temperature sensor Water temperature sensor Supplied air flow rate meter Inlet air temperature sensor Motion sensor	[83]
**2014**	Optimal chiller loading problem solved by swarm intelligence technique	Optimized setting	Power meter	[85]
**2015**	AI theory-based optimal control for improving the indoor temperature conditions and heating energy efficiency	Five control algorithms include Rule + ANN ANN + ANN Fuzzy + ANN ANFIS with two inputs + ANN ANFIS with one input + ANN	Temperature sensor Surface opening status detector	[86]
**2015**	Three houses with wireless sensors for detecting use occupancy and activity patterns	Optimized setting and predictive control	Thermocouple array Microphone Hygro sensor CO_2_ and air quality detector Ultrasonic sensor	[89]
**2016**	Model-based predictive control for the set point optimization of an HVAC system	Model-based predictive control	Temperature sensor Building energy analysis model with heat and moisture transfer through a wall	[92]
**2016**	Multi-objective control and management of a smart building	Optimized setting	Temperature sensor CO_2_ concentration detector Power meter	[93]
**2017**	Deep reinforcement learning for building HVAC control	Optimized setting	Temperature sensor Energy plus building model	[97]
**2018**	AI enhanced air conditioning comfort by Ambi Climate	Optimized setting	Temperature sensor Humidity sensor Sunlight sensor Geolocation by users’ mobile phone	[110]
